# Effect of a Dietary Supplement Combining Bioactive Peptides and Magnesium on Adjustment Disorder with Anxiety: A Clinical Trial in General Practice

**DOI:** 10.3390/nu14122425

**Published:** 2022-06-10

**Authors:** Sarah Oddoux, Paul Violette, Jeanne Cornet, Julie Akkoyun-Farinez, Michel Besnier, Antoine Noël, Frédéric Rouillon

**Affiliations:** 1 Laboratoire Dielen, 50110 Cherbourg-en-Cotentin, France; sarah-oddoux@dielen.fr (S.O.); paul-violette@dielen.fr (P.V.); 2 Euraxi Pharma, 37300 Joué-lès-Tours, France; j.cornet@euraxipharma.fr (J.C.); j.akkoyun-farinez@euraxipharma.fr (J.A.-F.); 3 Centre Médical Thémis, 50100 Cherbourg-en-Cotentin, France; mbesnier50@gmail.com; 4 Hôpital Ste Anne, 75014 Paris, France; f.rouillon@ghu-paris.fr

**Keywords:** anxiety, stress, bioactive peptide, magnesium, dietary supplement

## Abstract

Anxiety is a high frequency disorder in the general population. It is usually treated with benzodiazepines, which cause side effects and a dependence that could make withdrawal difficult. Alternative treatments are therefore needed to reduce the use of anxiolytics, particularly for adjustment disorder with anxiety. An observational, multicentre, prospective, longitudinal study has been conducted by general practitioners and one gynaecologist to evaluate the efficacy of a dietary supplement on adjustment disorder with anxiety (*Stress 2* study). Patients diagnosed as anxious with a score of ≥20 on the Hamilton Anxiety Rating Scale (Ham-A, first visit on Day 0 (V0)) were offered a 28-day treatment with a dietary supplement formulated with bioactive peptides from a fish protein hydrolysate (Gabolysat^®^), magnesium and vitamin B6. At the second visit (V1), the Ham-A Rating Scale, the Patient Global Impression scale (PGI) and the Clinical Global Impressions scale (CGI) were administered. A 50% reduction in the Ham-A score, was achieved for 41.9% of the patients. The mean Ham-A score decreased by 12.1 ± 5.7 points (*p* < 0.001) between V0 (25.6 ± 3.8) and V1 (13.6 ± 6.0). Furthermore, according to the CGI scale, the anxiety of 75.3% of patients improved significantly and very significantly, with limited side effects and a negligible rebound effect. In conclusion, adjustment disorder with anxiety seems to be effectively managed by an alternative and safer solution than benzodiazepines.

## 1. Introduction

Anxiety is defined as a mental state of unrest and agitation, a feeling of indefinable insecurity, a fear without an object. Its intensity is situated between worry and anguish, the latter associating somatic signs of oppression and constriction. Anxiety is distinct from depression, although the two conditions are often concurrent. Anxiety may or may not be associated with a somatic or mental illness. According to the two major classifications of psychiatric diagnoses (the World Health Organization’s International Classification of Diseases, version 10 (ICD-10), and the American Psychiatric Association’s Diagnostic and Statistical Manual of Mental Disorders, version 5 (DSM-5)), anxiety disorders are components of several syndromes [1]:

- Generalized anxiety disorder: a state of permanent anxiety and excessive worry lasting at least 6 months, unrelated to a specific object or situation. In Western countries, it affects 4% of the population, especially women. It is accompanied by motor tension and hypervigilance (concentration difficulties, sleep disorders, irritability). It has a strong impact on the person’s life and is often associated with depression;
- Panic attack: sudden onset of intense fear, a feeling of imminent disaster and loss of control, unrelated to an objective vital risk;
- Panic disorder: repetition of panic attacks, accompanied by the fear of being afraid;
- Phobic disorder: unreasonable, intense, object- or situation-specific fear, considered pathological when it affects the person’s life;
- Obsessive-compulsive disorder: fears that invade the mind permanently and become obsessive fears;
- Post-traumatic stress disorder: development of a pattern of symptoms following exposure to a traumatic event. These symptom types include re-experiencing the traumatic event, avoidance of stimuli associated with the trauma, persistent negative alterations in cognition, a numbing of general responsiveness, and increased symptoms of arousal.

The DSM-5 also defines adjustment disorders as a mental illness characterized by the development of clinically relevant emotional or behavioural symptoms (e.g., anxiety or depression) resulting from experiencing a psychosocial stressor(s). It also specifies that the symptoms do not represent normal bereavement [1]. Symptoms must occur within 3 months of the stressor(s). These disorders are to be distinguished from disorders related to post-traumatic stress or bereavement. They disappear spontaneously within 6 months after the stress has ended, but may persist if the stress is chronic or has long-term consequences. Adjustment disorder with anxiety is the most common of these adjustment disorders. Its predominant manifestations are symptoms such as nervousness, worry or agitation [2,3,4].

The management of anxiety disorders varies according to the situation. Treatment may include lifestyle advice (reduction of stimulants, physical activity), relaxation, psychotherapy and medication (most often benzodiazepines) [5]. The management of adjustment disorders is mainly based on psychotherapy and pharmacotherapy, although they have not really been proven to be effective. In current practice, benzodiazepines are most often prescribed, despite their side effects on cognitive function and the risks of dependence with long-term use [4,6].

Several studies carried out over the last decades, show that protein lysates from various food sources, in addition to their nutritional properties, have several biological activities. Indeed antioxidant, antimicrobial, hypotensive, anticoagulant, anti-cholesteric, hypoglycaemic and antitumor properties have been demonstrated (for review see [7]). These effects are associated with biopeptides of 2 to 50 amino acids, present in protein hydrolysates. Biopeptides possess beneficial pharmacological properties, due to their amino acid sequence and composition [8]. The beneficial effect of fish hydrolysate on anxiety has been shown in animal models [9,10,11,12]. In rats, Gabolysat^®^, a fish protein hydrolysate, exhibits effects similar to those of diazepam (an anxiolytic drug of the benzodiazepine family) on the pituitary–adrenal axis, on sympathoadrenal activity, as well as on the gamma-aminobutyric acid (GABA) content of the hippocampus and hypothalamus under resting and stress conditions [9]. In addition, still in a rodent model, Gabolysat^®^ at a dose of 15 mg/kg (equivalent to 2.43 mg/kg in humans, according to the guide established by Nair et al. [13], i.e., 170 mg for a 70 kg man) was as effective as diazepam in reducing anxiety in a conditional burying test within 30 min after ingestion. A clinical study versus placebo of school examination anxiety in 70 students treated with a fish lysate for 3 weeks suggests that this food supplement has an effect on this condition. No adverse effects were reported and the effect seems to be maintained for several weeks after stopping the treatment [14].

Magnesium (Mg) is the fourth most abundant cation in the body and the second most abundant in intracellular fluid. It is a cofactor for some 350 cellular enzymes, many of which are involved in energy metabolism. The recommended daily intake of Mg is 375 mg [15]. Mg is an inhibitor of the NMDA (N-methyl-D-aspartate) receptor [16] and an agonist of the GABAA (γ-aminobutyric acid A) receptor [17]. Preclinical studies have shown that a concentration of 20 mg/kg of Mg has an anxiolytic effect in mice [18]. The GABA_A_ receptor and the NMDA receptor are involved in the anxiolytic activity of Mg in mice [19]. Despite preclinical evidence for a role for Mg in the treatment of anxiety, clinical trials investigating its effect on this condition are limited. De Souza et al. showed that a one-month treatment of 200 mg Mg/day combined with 50 mg vitamin B6/day reduced anxiety associated with premenstrual symptoms [20]. Treatment with 124 mg Mg oxide combined with plant extracts was more effective than placebo in patients with mild to moderate long-term generalised anxiety disorder [21]. In addition, a very recent study on depression showed that taking 248 mg of MgCl_2_ for 6 weeks has a clinically significant beneficial effect on generalized anxiety disorder [22].

The majority of Mg salts are poorly absorbed and high doses can induce adverse effects in the digestive tract (diarrhoea in particular) [23]. Mg supplements are available as inorganic or organic salts, depending on their natural or induced association with mineral salts (chloride, oxide, sulphate) or organic molecules (citrate, lactate, amino acids). The type of compound to which Mg is bound affects the way in which Mg is absorbed. In general, organic salts have a better bioavailability than inorganic salts despite a lower elemental Mg content [24]. Mg bisglycinate is considered a preferred source of Mg. Indeed, several studies have shown a high bioavailability [25] and an efficacy at low dosages, which surpasses that observed for inorganic salts and for organic salts. The laxative effect of Mg bisglycinate is reduced compared to other sources of Mg because the element is present in its neutral form and does not induce an increase in osmotic pressure in the gut, which causes diarrhoea. Furthermore, the presence of glycine, an amino acid with a cytoprotective role that participates in several functions in the central nervous system and energy production, also has beneficial effects (for review see [26]). The same tolerance is expected from a marine Mg oxide chelated with amino acids from a rice protein hydrolysate that has a bioavailability similar to Mg bisglycinate [24].

Vitamin B6 acts as a cofactor for over 100 enzymes, including enzymes involved in the metabolism of neurotransmitters, such a serotonin or GABA [27]. It has also been shown that high doses of vitamin B6 could increase the Mg level in red blood cells [28]. In addition, a cross-sectional study showed that lower vitamin B6 intakes are associated with higher risks of depression and anxiety [29]. Several clinical trials have shown that the association of Mg and vitamin B6 was superior to magnesium alone, for example for premenstrual syndrome [20] or severe to extremely severe stress [30].

A randomised controlled clinical trial evaluating the efficacy of the combination of Mg and vitamin B6 in the management of anxiety [31,32] tested a 6-week treatment of 192 mg lactate with 20 mg vitamin B6 versus 40 mg buspirone. Treatments were administered after one week of placebo administration to exclude patients with a placebo effect. Ninety-nine patients (38.4% men and 61.6% women) with generalised anxiety disorder were included. A decrease in the Ham-A score was observed in each group but no significant difference was found between the groups [32]. These results confirm the interest of the combination of Mg and vitamin B6 in the management of anxiety since they present an effectiveness superior to placebo but equivalent to the drugs usually prescribed in the management of anxiety such as benzodiazepines or buspirone.

As the efficacy of Mg and vitamin B6 has never been assessed combined with an anxiolytic fish protein hydrolysate it was necessary to conduct a clinical trial. Therefore, this observational study, *Stress 2*, evaluated the effect and tolerability of a dietary supplement (Magzen^®^) that combines the fish hydrolysate Gabolysat^®^, with two forms of Mg with high bioavailability (Mg bisglycinate and a rice proteo-chelate of Mg) and with vitamin B6, on the anxiety level of patients suffering from adjustment disorder with anxiety. Individually, these compounds have an anxiolytic effect, therefore we explored whether their combination had a potential synergistic action and could become a promising strategy for the treatment of anxiety.

## 2. Materials and Methods

### 2.1. Population

The target population for this clinical study was patients aged 18–70, suffering from adjustment disorder with anxiety and with a Ham-A score ≥ 20. It corresponded to patients who, according to the doctor, require the prescription of a non-medicinal anxiolytic treatment. Patients with anxiety of more than 3 months duration, or with other mental illness, or who had already been treated for their anxiety in the previous 3 months, were excluded from the study population, as they were likely to receive a more appropriate treatment than the study treatment.

### 2.2. Objectives of the Study

The main objective of the study was to evaluate the effect of the food supplement Magzen^®^ for four weeks in the treatment of adjustment disorder with anxiety. The secondary objectives of the study were to describe the evolution of the anxiety components and of the symptoms, the tolerability of the treatment, the compliance with the treatment and the rebound effect on anxiety in the week following the cessation of treatment.

### 2.3. Study Design and Tested Dietary Supplement

This is an observational, multicentric, prospective, longitudinal study carried out in metropolitan France by 25 general practitioners and one gynaecologist. Out of the 26 physicians, 17 investigators were active (at least 1 patient included and up to 10).

**Figure 1:**
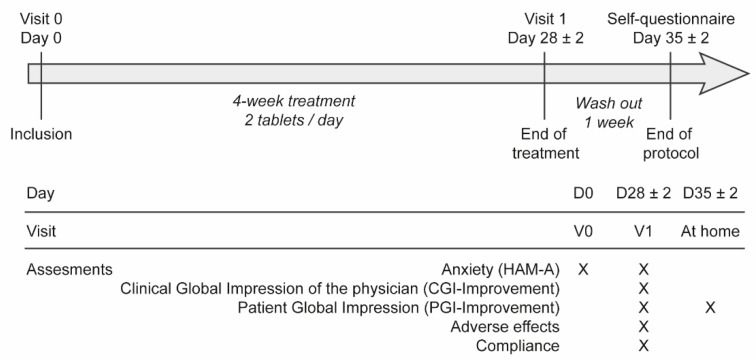
Treatment protocol. The “×” indicates the visits during which different tests were administered to patients. Text in italics refers to the length and treatment phases between the visits.

Each patient had to be followed for a period of 5 weeks (Figure 1), with two visits to the general practitioner or gynaecologist: an inclusion visit at Day 0 (V0) and a visit at Day 28 ± 2 days (V1). Finally, a self-assessment questionnaire had to be completed 7 days after the last day of treatment.

At the initial visit on Day 0 (V0), patients were included by their general practitioner or specialist, after checking the inclusion/exclusion criteria, being informed about the study and expressing their consent to participate. During this consultation, the following was completed: the Hamilton Anxiety Rating Scale (Ham-A), socio-demographic and clinical data, history of the anxiety disorder, medical history, previous treatments over the last 3 months and current treatments. At the end of the consultation, the physician provided the dietary supplement to the patient (1 box of the dietary supplement containing 60 tablets), who had to take 2 tablets/day from Day 1 to Day 28 ± 2, in the morning with breakfast, without chewing them. Two tablets of the dietary supplement (Magzen^®^, Laboratoire Dielen^®^) provide 300 mg of Mg (270 mg proteo-chelated marine oxide Mg + 30 mg bisglycinate Mg), 200 mg of fish protein hydrolysate Gabolysat^®^ and 1.4 mg of vitamin B6 (pyridoxal chlorate).

At the visit on Day 28 ± 2 days (V1), the patient had to return the empty, partially used or complete blisters. During this consultation, the following were completed: the Ham-A Rating scale, the Clinical Global Impression, Improvement (CGI-I) scale, the CGI, Efficacy Index scale (CGI-E), the self-assessment Patient Global Impression, Improvement (PGI-I) scale (depending on the patient’s response), the self-assessment Girerd questionnaire (depending on patient response), the number of unused tablets and safety information.

At the end of this visit, the physician was asked to give the patient a PGI-I questionnaire on the evolution of his/her anxiety, which he/she was asked to fill in 7 days later to assess a possible rebound effect of the anxiety when the treatment was stopped. However, if the physician considered that it was required to extend the treatment beyond 30 days, the self-assessment questionnaire was not given to the patient.

### 2.4. Inclusion/Exclusion Criteria

A patient was eligible for inclusion if he/she met all of the following criteria: age ≥ 18 and ≤70 years; patient with adjustment disorder with anxiety; Ham-A score ≥ 20 at inclusion; recent anxiety (duration ≤ 3 months) not managed by pharmacological or psychological treatment; disorder requiring, according to the doctor, prescription of an anxiolytic treatment by non-medicinal treatment; patient able to understand the information related to the study, to read the information leaflet and who consented to participate in the study.

A patient with at least one of the following criteria could not be included in the study: a patient with another type of anxiety disorder (generalized anxiety disorder, obsessive-compulsive disorder, acute post-traumatic stress disorder), major depressive syndrome or any other mental pathology; anxiety related to bereavement; anxiety experienced for more than 3 months; patient treated with psychotropic drugs or psychotherapy for anxiety in the last 3 months; alcohol or drug dependence; excessive consumption of coffee or caffeinated beverages (more than 10 mg/kg of caffeine, about 6 average cups of coffee per day for a 60 kg adult); excessive smoking (more than one pack per day); a pregnant or breastfeeding women; intolerance or allergy to any of the components of the treatments under study and, in particular, fish allergy; patients with severe somatic pathologies (e.g., severe renal, hepatic or cardiac insufficiency, severe endocrine or metabolic disorder, stroke, progressive cancer...); patients participating in another clinical trial, or in a period of exclusion from another clinical trial.

### 2.5. Evaluation Criteria

The primary endpoint of the study was the number (%) of patients at V1, i.e., with a decrease in Ham-A score ≥ 50%. The Ham-A scale consists of 14 items measuring the severity of anxiety. Each item is scored from 0 (absent) to 4 (severe) [33]. The total score can vary from 0 to 56. A score below 17 suggests mild anxiety, between 18 and 24 suggests mild moderate anxiety, between 25 and 30 suggests moderate to severe anxiety and above 30 suggests severe anxiety.

Secondary endpoints: Ham-A score at V1; Ham-A subscores at V1; PGI-I scale (8 points) at V1, measuring overall improvement since V0 (0: no opinion; 1: very much improved, 2: much improved, 3: minimally improved, 4: not at all improved, 5: minimally worsened, 6: much worsened, 7: very much worsened); CGI-It scale at V1 (8-point Likert scale) (0: no opinion; 1: very much improved, 2: much improved, 3: minimally improved, 4: not at all improved, 5: minimally worsened, 6: much worsened, 7: very much worsened); CGI-E at V1 (16-point Likert scale giving a combined measure of the main clinical effect and side effects); Girerd score at V1 (6-question assessment of compliance. 0 “Yes” answers: good compliance; 1 or 2 “Yes” answers: minor non-compliant patient; 3 to 6 “Yes” answers: major non-compliant patient); the ratio between the number of tablets actually taken and the number of tablets theoretically taken according to the prescription (compliance assessment); total number of adverse events (AEs) and number (%) of patients with at least one AE by type, relationship to treatment, severity and consequence on continuation of treatment; number (%) of patients who reported a worsening of their anxiety between V1 and Day 35 ± 2 (potential rebound effect on stopping treatment) on an 8-point Likert scale (0: no opinion; 1: very much improved, 2: much improved, 3: minimally improved, 4: not at all improved, 5: minimally worsened, 6: much worsened, 7: very much worsened) filled in by the patient at home, 7 days after the last day of the treatment.

### 2.6. Statistical Methods

#### 2.6.1. General

The analysis was carried out with SAS^®^ software version 9.4 (SAS Institute, Cary, NC, USA). The inferential analyses were preceded by descriptive analyses. Quantitative variables were described by the number of values filled in, the number of missing data (MD), the mean, the standard deviation, the median, the 1st and 3rd quartile, and the minimum and maximum. Categorical variables were described by the number of values filled in, the number of missing values, the frequency and the percentage per modality. The first order risk (α) was set at 5% in a two-sided situation.

#### 2.6.2. Rationale for the Number of Subjects

The primary endpoint of the study was to determine the percentage of patients who showed a decrease in their Ham-A score ≥ 50%, considered responders to treatment. Data from the literature show a response rate of 40% with placebo [21]. With an expected response rate of 60%, a two-sided alpha risk of 5% and a power of 90%, it was necessary to analyse 93 patients to be able to describe this rate with an accuracy of 10%. Taking into account a 15% rate of non-analysable forms, 107 patients needed to be included.

#### 2.6.3. Analysed Population

The “analysed” population corresponded to all patients included in the study, without major deviations from the protocol (non-compliance with inclusion/non-exclusion criteria and missing primary endpoint assessment, out of time between V0 and V1, no evidence of treatment, treatment prohibited) and with an assessment of the primary endpoint. All analyses were performed on the analysed population. To be in accordance with standard medical practice, minor deviations to the protocol were accepted (V1 between 20 and 41 days after V0/2nd visit (V2) between 25 and 46 days after V0).

#### 2.6.4. Imputation of MD

In general, no imputation of MD was performed. Overall remarks in case of multiple ticking of an item:

- If more than one box was ticked to answer an item in the questionnaires used in the study, the most severe answer (worst choice) was retained for all analyses
- No estimates of missing questionnaires were made. If the follow-up questionnaire was missing, the data was considered missing. If the patient exits the study due to lack of efficacy, then the treatment will be considered as failed. Concerning the primary endpoint (Ham-A score):
- If more than 20% of the items on the Ham-A scale were missing, the total score was considered missing
- If less than 20% of the items were missing, a correction was applied based on the proportionality rule:
  (1) $$\frac{\text{total score of missing items}\;\times \;\text{total number of items}}{\mathrm{number of items filled in}}$$
- Missing Ham-A scores were imputed according to the baseline-observation-carried-forward (BOCF) model, which consists of replacing the missing value with the baseline value for the sensitivity analysis of the primary endpoint.
- Incomplete dates: no imputation on missing dates was performed, except for the date of initial diagnosis for which, if the day was missing, it was estimated as the 1st of the month and if the month was missing, it was estimated as January.

#### 2.6.5. Sensitivity Analyses of the Primary Objective

The percentage of responders to treatment was calculated with a 95% confidence interval (95% CI) on the total population with replacement of MD at V1 by the BOCF method. A second sensitivity analysis of the primary endpoint was performed on the total population.

## 3. Results

### 3.1. Study Population

One hundred and ten patients were included in the study from 8 November 2019 to 6 April 2021 (total population). Among the 110 patients, nine patients did not complete the study according to the protocol for the following reasons:

- Not returned at V1 (lost to follow-up): three patients;
- Premature discontinuation due to AEs: four patients;
- Premature discontinuation of treatment due to the need to introduce an antidepressant: two patients.

**Figure 2:**
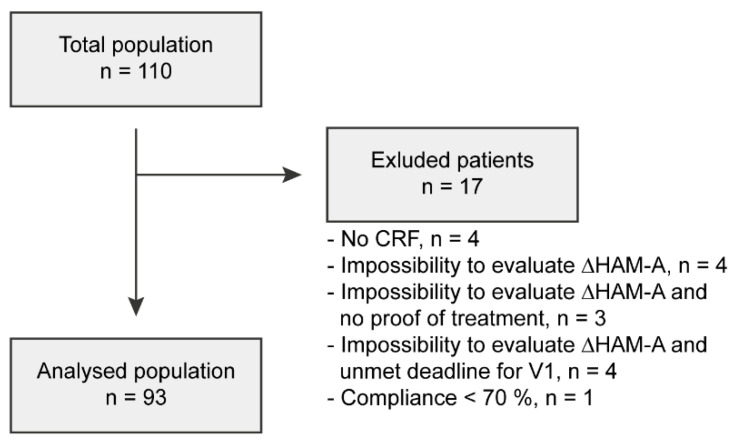
Number of patients included in the study, excluded and analysed. CRF: case report form.

Seventeen patients were excluded from the total population for major deviations from the protocol described in Figure 2. Thus, the analysed population comprised 93 patients. The description of the populations is shown in Figure 2.

The characteristics of the total population are similar to those of the analysed population. Therefore, only the characteristics of the analysed population are presented. The characteristics are summarized in Table 1. Of the 93 patients of the analysed population, 17.2% were men and 82.8% were women. On average, the patients were 49.6 ± 13.1 years old, weighed 69.4 ± 13.0 kg and were 165.4 ± 6.8 cm tall with a mean BMI of 25.35 ± 4.41 kg/m^2^. The majority of the patients (67.4%) were living with a partner and about a quarter of the patients had no children (23.9%), a quarter had one child, almost a third had two children (34.1%) and 17% had three or more children.

Concerning the professional situation of the patients, the most represented socio-professional category was that of employees (39.1%) and the majority of patients had a stable job (57.5%). Almost two thirds of the patients drank alcohol occasionally (66.3%). Regarding the consumption of caffeinated drinks, 24.0% of the patients did not drink caffeinated beverages and 68.5% drank less than three cups per day. The vast majority of patients never smoked (70.5%) or no longer smoked (17.0%). Slightly more than two-thirds of the patients (68.7%) were physically active and 41.0% of the patients were physically active at least twice a week.

Half of the patients had at least one medical history (50.5%). The most common medical history was medical and surgical procedures (10.8% of patients), nervous system conditions (8.6%) and vascular conditions (8.6%) (Table 2). At the time of inclusion, 28% of patients had ongoing treatment, mainly for cardiovascular disease.

Patients had been suffering from anxiety disorders for an average of 1.30 ± 0.73 months. Almost all patients had at least one identified stressor (90.2%). These were mainly family difficulties (54.2%) and/or professional difficulties (49.4%). Only 25.8% of the patients had suffered from anxiety episodes requiring treatment or psychotherapy before the current anxiety episode (7.5% of the patients in the analysis population had previously been treated with benzodiazepines, 7.5% with antidepressants, 7.5% with psychotherapy, 6.5% with homeopathy, 3.2% with phytotherapy/dietary supplements and 2.2% with anxiolytics other than benzodiazepines). The same patient could have received several different treatments.

### 3.2. Primary Objective Analyses

In the analysed population, 41.9% of the patients (39/93) meet the primary objective with a decrease in the Ham-A score of ≥50% (CI 95%: 31.9%–52.0%). The results of the sensitivity analysis, performed on the total population without imputing MD, differ little from those of the main analysis as 41.8% (12 MD) of patients in the total population had a decrease in their Ham-A score of ≥50% (CI 95%: 32.1%–51.6%). The bias on the primary endpoint due to the exclusion of patients appears to be minimal; with imputation of MD, the proportion of patients in the total population with a decrease in their Ham-A score of ≥50% was slightly lower (39.4% (CI 95%: 29.5%–49.4%)).

In order to understand more precisely the patients’ experience and the evolution of the components of anxiety, we performed secondary analyses.

### 3.3. Secondary Analyses

#### 3.3.1. Evolution of the Ham-A Score and Subscores

**Figure 3:**
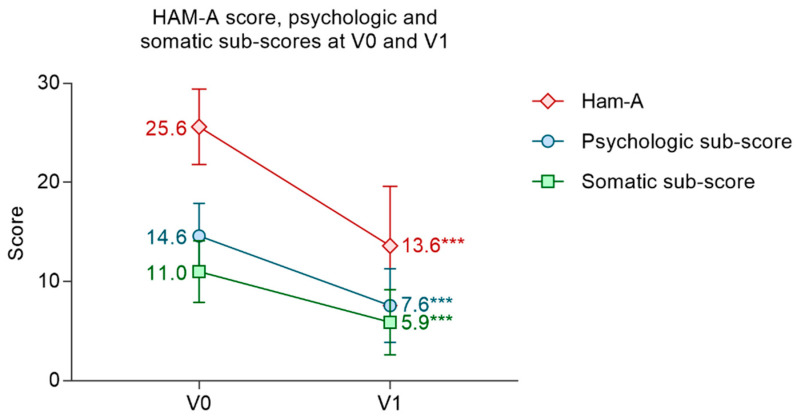
Evolution of the Ham-A score and psychologic and somatic subscore between V0 and V1. Ham-A variation: Student’s *t*-test; psychological subscore variation: Wilcoxon signed-rank test; somatic subscore variation: Student’s *t*-test. Mean ± SD. *** *p* < 0.001.

At inclusion, patients had a mean Ham-A score of 25.6 ± 3.8. The mean psychologic subscore was 14.6 ± 3.3 and the mean somatic subscore was 11.0 ± 3.1. The mean Ham-A score decreased significantly by 12.1 ± 5.7 points (*p* < 0.001) between V0 and V1 (13.6 ± 6.0). The mean psychologic and somatic subscores both decreased significantly between V0 and V1, respectively, by −7.0 ± 3.9 points (*p* < 0.001) and −5.1 ± 3.1, (*p* < 0.001) (Figure 3).

**Figure 4:**
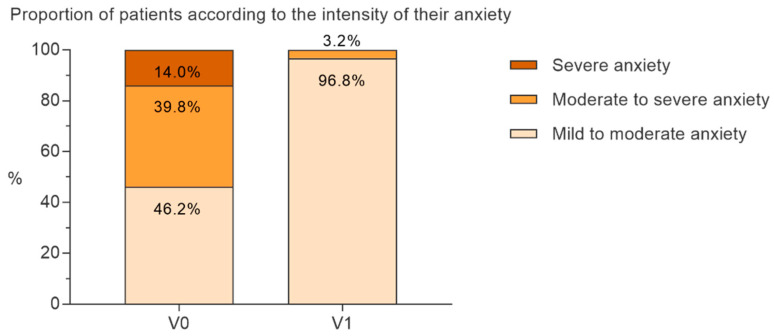
Percentage of patients with severe (Ham-A > 30), moderate (25 ≤ Ham-A ≤ 30) or mild anxiety (Ham-A ≤ 24) at V0 and V1.

At V0, 46.2% of the patients had mild to moderate anxiety (Ham-A ≤ 24), 39.8% had moderate to severe anxiety (Ham-A: 25–30) and 14.0% had severe anxiety (Ham-A > 30). At V1, almost all (96.8%) patients had mild to moderate anxiety, only 3.2% had moderate to severe anxiety, and no patient suffered from severe anxiety anymore (Figure 4).

#### 3.3.2. Clinical Improvement According to Physicians (CGI-I Score) and to Patients (PGI-I Score)

**Figure 5:**
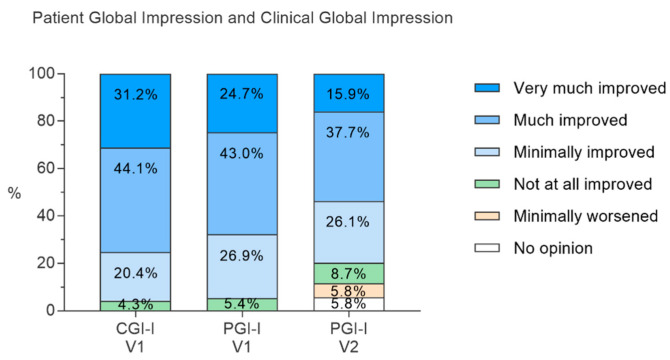
Patient’s and clinician’s global impression on patient’s anxiety improvement at the end of the treatment (V1) and 7 days after (V2), Patient Global Impression, Improvement (PGI-I), Clinical Global Impression, Improvement (CGI-I).

The responses to the CGI-I and PGI-I questionnaires are shown in Figure 5. At the V1 visit, physicians considered that, at the end of the treatment, 75.3% of the patients improved (CGI-I V1, much or very much improved). No worsening of anxiety was reported by the physicians. Similarly, at V1, 67.7% of the patients considered that their anxiety had improved following treatment (PGI-I V1, much or very much improved). No patient considered that their anxiety had worsened. Regarding the evolution of anxiety seven days after the end of the treatment, among the patients who met the time limit of 25–46 days after the V0 visit (*n* = 70, 1 MD), half of the patients (53.6%) considered that their anxiety had improved on the PGI-I scale (much or very much improved). Only 5.8% of patients experienced a rebound effect, i.e., a worsening of their anxiety seven days after stopping the supplementation. However, this worsening was described as minor by all patients concerned.

#### 3.3.3. Analysis of Compliance

Based on the number of tablets returned, or the number of tablets reportedly not taken by the patient, as no tablets were returned, patients took their treatment correctly. Indeed, the average percentage of compliance was 103.5% (min: 87.9%–max: 142.9%, 16 MD). According to the Girerd score (3 MD), two thirds of the patients demonstrated good compliance and about one third (32.2%) demonstrated minor non-compliance. The most compliant patients responded better to the treatment than the others: 51.6% of the good compliance patients had a decrease of more than 50% in their Ham-A score compared to 24.1% of the non-compliant patients (*p* = 0.017).

#### 3.3.4. Analysis of the Therapeutic Effect with Regard to Tolerance

**Figure 6:**
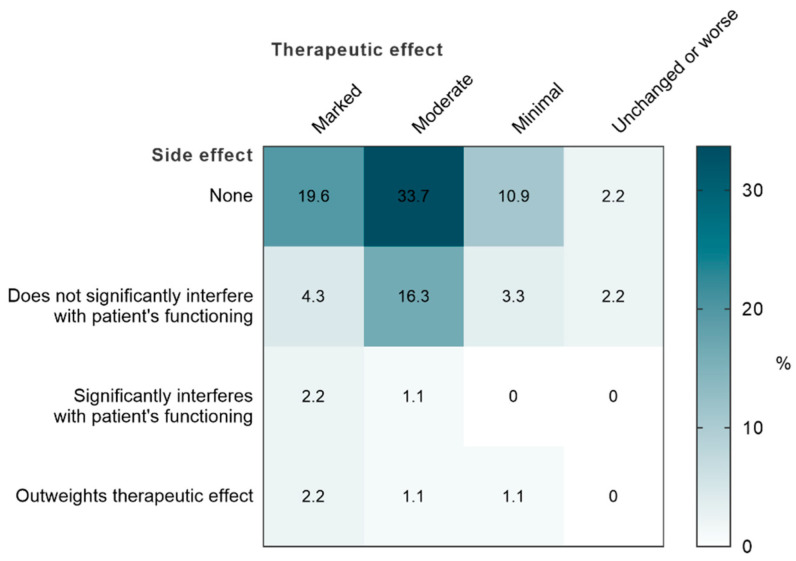
Clinical Global Impression—Efficacy Index.

At V1, for almost three quarters of the patients (73.9%), the physicians considered that the dietary supplement had a moderate or marked therapeutic effect without any side effect or without side effects having major interference on the patient’s activities (Figure 6). The majority of adverse events possibly related to the supplementation were not serious, though some led to a premature cessation of the treatment (Table S1).

## 4. Discussion

One hundred and ten patients suffering from adjustment disorder with anxiety were included in the *Stress 2* study. The majority of the patients were women (82.8%), which is explained by the fact that women are more affected by anxiety disorders than men (two times more in the general population) [34]. At inclusion, patients had a mean Ham-A score of 25.6 ± 3.8, representing moderate to severe anxiety (Ham-A: 25–30). Among the analysed patients, 41.9% (IC 95%: 31.9%–52.0%) showed a significant decrease ≥50% in their Ham-A score within the 28 days course of the treatment. The mean Ham-A score decreased significantly by −12.1 ± 5.7 points (*p* < 0.001) between V0 (25.6 ± 3.8) and V1 (13.6 ± 6.0), i.e., a reduction of 47.3%. The average psychologic and somatic subscores both decreased significantly between V0 and V1, respectively, by −7.0 ± 3.9 points (*p* < 0.001) and −5.1 ± 3.1, (*p* < 0.001). These results suggest a harmonious action of the dietary supplement on the two components of anxiety.

The absence of a comparator group does not allow a conclusion to be drawn on the efficacy of the treatment, as the observed effect can be attributed to the natural course of the patients. However, the fact that no deterioration in clinical condition was observed, either by the patients or by the physicians, argues in favour of the efficacy of the treatment. In addition, a clinical study on anxiety during school examinations, conducted on 70 students treated with Gabolysat^®^ or a placebo for 3 weeks, suggests that the food supplement is superior to placebo for anxiety from week 2 [14].

The results of the *Stress 2* study should also be put into perspective with those of the randomised double-blind study by Nguyen et al. [35]. This study compared two anxiolytics (lorazepam and etifoxin) in the treatment of adjustment with anxiety. One hundred and ninety-nine patients were included. Ninety-three patients received etifoxin 150 mg daily for 28 days and 96 patients received lorazepam 2 mg daily for 28 days. The total study population had quite similar characteristics to those of the *Stress 2* study: mean age 43 years (18–68 years), 66.1% female, family-related stressors in 40.7%, occupational stressors in 29.6% and health-related stressors in 8.5% of patients. The mean Ham-A score at inclusion was 25.5 (20–38), i.e., almost the same as in the *Stress 2* study (25.6).

- The responder rate (decline of ≥50% in Ham-A score) after 28 days of treatment was 72% in the etifoxin group and 56% in the lorazepam group. The responder rate in the *Stress 2* study was 41.9%.
- The mean Ham-A score at Day 28 was 11.4 ± 5.9 in the etifoxin group (versus 25.2 ± 3.5 at Day 0) and 12.2 ± 6.4 in the lorazepam group (versus 25.6 ± 4.2 at Day 0), a reduction of 54.6 ± 23.5% and 52.3 ± 24.2%, respectively. In the *Stress 2* study, the reduction in Ham-A score was 47.3%.
- Clinical improvement according to the CGI score (much or very much improved) was 73.3% in the etifoxin group and 57.1% in the lorazepam group. This improvement was seen in three quarters of the patients in the *Stress 2* study.

Although the supplementation does not reduce anxiety to the same extent as anxiolytics, its efficacy is not negligible compared to the results of the Nguyen et al. study [35], particularly with regard to its tolerability (see below). The PGI, which assesses patients’ feelings (not collected in the Nguyen et al. study), supports these encouraging results for the tested dietary supplement.

Concerning the evolution of anxiety seven days after the end of the treatment, only 5.8% of the patients experienced a rebound effect, i.e., a worsening of their anxiety seven days after stopping the supplementation. However, this worsening was described as minor by all the patients concerned. This is consistent with the observation from a previous clinical trial on a different formulation of Gabolysat^®^, where the 1-week wash-out between the supplement and the placebo was null because of an unanticipated effect of the supplement [14]. This rebound phenomenon, observed for benzodiazepines, therefore, does not seem to be very frequent in our study, and does not seem to be associated with a withdrawal syndrome (the worsening would be more marked if this were the case). In comparison, rebound at Day 35 (7 days after stopping treatment) occurred in 9.1% of patients in the lorazepam group in the study by Nguyen et al. [35].

In rats, Gabolysat^®^ showed an anxiolytic activity comparable to that of diazepam as early as 30 min after its administration as well as 60 min after. Therefore, Gabolysat^®^ has the advantage of acting rapidly and durably on anxiety [36]. The efficacy of the supplement could be explained by the combination of active ingredients that act on the GABAergic system. Gabolysat^®^ has been shown to increase the GABA content in the hypothalamus in rats [9], Mg has been shown to stimulate GABA release (for review, see [37]), and finally, with vitamin B6 being the cofactor of the glutamic acid decarboxylase [38], its deficiency in rats leads to a decrease in GABA in the cerebellum [27]. According to a secondary analysis of the second individual and national study on food consumption study (INCA2 study [39]), the French population aged 18 to 75 have an inadequate intake of vitamin B6 of 13.9 to 20.1% of the recommended amount. This inadequacy rises from 67.4 to 76.6% for Mg intakes [40]. Therefore, it could be of interest to try supplementation with Mg and vitamin B6 for anxious patients who could be deficient in these nutrients. Hypothetically, the fish protein hydrolysate of the supplement tested in the *Stress 2* study could amplify the effects of Mg and vitamin B6 on the GABAergic system. A comparison between groups treated with Mg and vitamin B6 with or without Gabolysat^®^ could be interesting to test this hypothesis.

Regarding compliance, the most compliant patients responded better to treatment than the others; 51.6% of the good compliance patients responded well to treatment compared to 24.1% of the non-compliant patients (*p* = 0.017). These results are encouraging regarding the effectiveness of the dietary supplement in improving anxiety. However, they should be analysed with caution. Indeed, it is commonly accepted that compliance is generally associated with the degree of patient confidence in the treatment and therefore with the placebo effect. For example, patients who have the least trust in their doctor are more reluctant to take the treatment and tend to be less compliant. The same is true for patients who do not believe in the effectiveness of the treatment, who have side effects or who do not experience rapid improvement. On the contrary, patients who believe in the treatment or who see rapid effects are usually more compliant. This phenomenon explains a less pronounced placebo effect in non-adherent patients, a placebo effect that is difficult to assess in an open trial.

Reported adverse reactions were mainly non-serious and mainly gastrointestinal disorders. The giant urticaria was probably due to a previously undetected allergy to fish. These adverse reactions are similar to those of MagneB6^®^ which, according to the product’s Summary of Product Characteristics (SmPC), correspond to hypersensitivity, diarrhoea, abdominal pain, and skin reactions such as urticaria, pruritus, eczema and erythema. The food supplement was therefore generally well tolerated and better tolerated than benzodiazepines, which cause sedation, cognitive and psychomotor disturbances, and memory loss [41].

The dietary supplement tested in this study may be of interest in the management of anxiety disorders to avoid or delay the use of medication such as benzodiazepines, well known for their adverse effects.

## 5. Conclusions

The results of the *Stress 2* study suggest that supplementation formulated with a fish protein hydrolysate, Mg and vitamin B6 reduces adjustment disorders with anxiety while being fairly well tolerated, and without causing rebound phenomena upon discontinuation. The absence of a comparative group does not allow us to conclude regarding the effectiveness of the treatment compared to a placebo. However, given the clinical data in the literature on the Mg/vitamin B6 combination and the animal data for the protein hydrolysate, the food supplement appears to be an interesting therapeutic alternative, particularly to benzodiazepines, which cause numerous side effects, in the treatment of these disorders.

## Figures and Tables

**Table 1:** Description of the analysed population.

Parameters	Characteristics	Proportion
Sex	Male	16 (17.2%)
	Female	77 (82.8%)
Age (years)	Mean ± SD	49.6 ± 13.1
	Min; Max	21; 73 *
	(18–29) years	6 (6.6%)
	(30–45) years	27 (29.7%)
	≥45 years	58 (63.7%)
	MD	2
Weight (kg)	Mean ± SD	69.4 ± 13.0
	Min; Max	42; 100
	MD	3
Size (cm)	Mean ± SD	165.4 ± 6.8
	Min; Max	150; 182
	MD	2
BMI (kg/m^2^)	Mean ± SD	25.35 ± 4.41
	Min; Max	16.8; 35.9
	Underweight (BMI < 18.5)	2 (2.2%)
	Normal (18.5 ≤ BMI < 25)	41 (45.6%)
	Overweight (25 ≤ BMI < 30)	30 (33.3%)
	Obese (BMI ≥ 30)	17 (18.9%)
	MD	3
Marital status	Single	30 (32.6%)
	Couple	62 (67.4%)
	MD	1
Children	Mean ± SD	1.5 ± 1.2
	Min; Max	0; 5
	0 child	21 (23.9%)
	1 child	22 (25.0%)
	2 children	30 (34.1%)
	≥3 children	15 (17.0%)
	MD	5
Professional status	Stable employment	50 (57.5%)
	Precarious employment	5 (5.7%)
	In search of employment	2 (2.3%)
	On sick leave	3 (3.4%)
	Unemployed	27 (31.0%)
	MD	6
Socio-professional status	Craftsmen, traders and company managers	4 (4.3%)
	Executives and higher intellectual professions	11 (12.0%)
	Intermediate occupations	14 (15.2%)
	Employees	39 (39.1%)
	Workers	4 (4.3%)
	Not in the labour force (retired, etc.)	14 (15.2%)
	Unemployed	9 (9.8%)
	MD	1
Alcohol consumption	Never	27 (29.3%)
	Sometimes	61 (66.3%)
	≤2 glasses/day	4 (4.3%)
	MD	1
Caffein consumption	Never	21 (22.8%)
	<3 cups/day	63 (68.5%)
	<6 cups/day	8 (8.7%)
	MD	1
Smoking habit	Never	62 (70,5%)
	Former smoker	15 (17.0%)
	Smoker	11 (12.5%)
	MD	5
Physical activity	Never	26 (31.3%)
	<2 times/week	23 (27.7%)
	2 times/week	13 (15.7%)
	>2 times/week	21 (25.3%)
	MD	10

Mean ± standard deviation (SD). Missing data: MD. * Patients up to 73 years old were accepted in the analysed population (minor deviation).

**Table 2:** Description of medical history of anxiety disorders.

Parameters	Characteristics	Proportion
Age of anxiety disorder (months)	Mean ± SD	1.30 ± 0.73
	Min; Max	0.1; 3.1
	MD	5
At least one identified stressor(s)	No	9 (9.8%)
	Yes	83 (90.2%)
	MD	1
Identified stressors	Professional difficulties	41 (49.4%)
	Family difficulties	45 (54.2%)
	Health issues	10 (12.0%)
	Financial difficulties	7 (8.4%)
	Social conflict	2 (2.4%)
	Other	10 (12.0%)
Former anxiety episodes requiring treatment or psychotherapy	No	69 (74.2%)
	Yes	24 (25.8%)
Ham-A V0	Mean ± SD	25.6 ± 3.8
	Min; max	20; 37
Psychologic subscore V0	Mean ± SD	14.6 ± 3.3
	Min; max	9; 23
Somatic subscore V0	Mean ± SD	11.0 ± 3.1
	Min; max	4; 17

V0: visit on Day 0.

## Data Availability

The data presented in this study are available on request from the corresponding author.

## Supplementary Materials

The following are available online at https://www.mdpi.com/article/10.3390/nu14122425/s1, Table S1: Number and percentage of adverse events attributable to the food supplement in the safety population: at least one dose of food supplement (*n* = 103).
