# Supplementary material for: Effect of a Dietary Supplement Combining Bioactive Peptides and Magnesium on Adjustment Disorder with Anxiety: A Clinical Trial in General Practice
Source: Nutrients. 2022 Jun 10;14(12):2425. doi: 10.3390/nu14122425 (PMC9228954; doi:10.3390/nu14122425)
Supplement: Supplementary file 1 [file nutrients-14-02425-s001.zip › nutrients-1702016-supplementary.pdf]

Table S1. Number and percentage of adverse events attributable to the food supplement in the safety population: at least one dose of food supplement (n = 103).

		N events	N (%) patients
All		12	8 (7.8 %)
Gastrointestinal disorders	All	10	6 (5.8%)
	Diarrhoea	6	5 (4.9%)
	Abdominal distention	1	1 (1.0%)
	Abdominal pain	1	1 (1.0%)
	Dyspepsia	1	1 (1.0%)
	Gastroesophageal reflux	1	1 (1.0%)
Skin and subcutaneous tissue disorders	All	1	1 (1.0%)
	Giant urticaria*	1	1 (1.0%)
Psychiatric disorders	All	1	1 (1.0%)
	Insomnia	1	1 (1.0%)
